# Rotavirus Genomic RNA Complex Forms via Specific RNA–RNA Interactions: Disruption of RNA Complex Inhibits Virus Infectivity

**DOI:** 10.3390/v9070167

**Published:** 2017-06-29

**Authors:** Teodoro Fajardo, Po-Yu Sung, Cristina C. Celma, Polly Roy

**Affiliations:** Department of Pathogen Molecular Biology, Faculty of Infectious and Tropical Diseases, London School of Hygiene and Tropical Medicine, London WC1E 7HT, UK; ted.fajardo@lshtm.ac.uk (T.F.J.); po-yu.sung@lshtm.ac.uk (P.-Y.S.); cristina.celma@lshtm.ac.uk (C.C.C.)

**Keywords:** virus assembly, RNA interaction, rotavirus, *Reoviridae*, genome packaging

## Abstract

Rotavirus (RV), a member of the *Reoviridae* family, causes infection in children and infants, with high morbidity and mortality. To be viable, the virus particle must package a set of eleven RNA segments. In order to understand the packaging mechanism, here, we co-synthesized sets of RNA segments in vitro in different combinations and detected by two alternate methods: the electrophoretic mobility shift assay (EMSA) and the RNA-bead pull-down assay. We showed that viral positive-sense RNA segments interact with each other in a specific manner, forming RNA complexes, and that the RNA–RNA interactions followed a sequential order initiated by small RV segments. Further, we demonstrated that RNA complexes were perturbed by targeted specific antisense oligoribonucleotides (ORNs) complementary to short RNA sequences, indicating that the RNA–RNA interactions between different segments were sequence-specific. The same inhibitory ORNs also had the capability to inhibit virus replication. The combined in vitro and in vivo data inferred that RNA–RNA interactions and specific complex formation are essential for sorting different segments, possibly prior to, or during, genome packaging. As genome assembly is a universal requirement in the *Reoviridae* family, this work offers an approach towards a further understanding of the sorting and packaging mechanisms of RV and related dsRNA (double-stranded RNA) viruses.

## 1. Introduction

Rotavirus (RV) gastroenteritis is a highly contagious, acute viral disease that remains a major cause of childhood diarrhoeal disease worldwide, notably in developing countries. The high infant mortality rate associated with RV infections, and the global burden of RV disease, have driven the development of several live attenuated vaccines, which, while protective in developed countries, are less effective in developing countries where the RV infection is highly prevalent. RV is a non-enveloped virus (a member of the *Reoviridae* family) with three layers of capsid proteins enclosing the viral polymerase complex and a double-stranded (ds)RNA genome of 11 segments (S1–S11) with different sequences and lengths (small: S7–S11; medium: S4–S6; and large: S1–S3) [1,2,3]. Following entry into the host cell, like all other members of the family, the triple-layered RV particle (TLP) uncoats to generate a cytoplasmic double-layered particle (DLP) of two major structural proteins, VP6 (outer layer) and VP2 (inner layer), and the polymerase complex of VP1 and VP3, in addition to the viral genome. Upon entry, the DLP remains intact, but becomes transcriptionally active, synthesizing and extruding multiple copies of capped, positive-sense, single-stranded (ss)RNA transcripts of the 11 dsRNA segments [4,5]. The released transcripts, each with specific 5′ and 3′ untranslated regions (UTRs), serve as the templates for the synthesis of both viral proteins and RNA intermediates for the synthesis of progeny dsRNA genomic segments [2,6,7,8,9,10]. However, it is not exactly clear how the newly synthesized 11 RNA segments are sorted and packaged within an assembling viral capsid. Current accumulating data from other segmented RNA viruses suggest that packaging signals in the 5′- or 3′-UTRs are the key elements in the genome recruitment mediated by RNA–RNA interactions through the segment-specific packaging signals [11,12,13,14,15,16,17,18,19,20,21,22]. More recently, we have shown that the positive-sense ssRNA templates of bluetongue virus (BTV), also a member of the *Reoviridae* family with a genome of 10 dsRNA segments, are packaged prior to synthesis of genomic dsRNA [18,19,23]. Further, each BTV segment appears to possess RNA–RNA interacting signals that allows them to be sorted and packaged as an RNA complex, where the 3′-UTR of the smallest ssRNA segment plays a leading role in the RNA assembly [18,19]. It is hypothesized that the packaging of 11 genomic segments of RV would follow similar mechanisms as for BTV.

To investigate whether RV follows a similar packaging process, a series of RNA–RNA interaction assays, focusing on the three smallest RV genome segments (S9–S11), were undertaken by in vitro co-synthesis. Intermolecular interactions were initially observed between segments S9, S10 and S11, when analyzed by an electrophoretic mobility shift assay (EMSA). The RNA complexes among smaller segments were also maintained when larger RNA segments were added to the combinations (e.g., S8) indicating that additional RNA segments probably facilitated a larger complex formation through multipoint RNA–RNA interactions. Further, we found that oligoribonucleotides (ORNs) targeting putative RNA–RNA interacting sites of S10 and S11 segments inhibit the RNA complex formation in vitro, and the same ORNs could inhibit virus replication in cell culture.

As protein synthesis was unaffected, we predict that the ORNs disrupt segment sorting and packaging. This indicates that the formation of the genomic RNA complex is dependent on specific RNA–RNA interactions mediated by the 3′-UTR sequence. This study further demonstrates how ORNs can be used to dissect mechanisms of virus replication and RNA–RNA interactions.

## 2. Materials and Methods

### 2.1. Cells and Virus

The MA104 cell line (American Type Culture Collection, DC, USA) was maintained in minimum essential medium (MEM; Sigma, St. Louis, MO, USA) supplemented with 10% (*v*/*v*) fetal bovine serum (Invitrogen, Carlsbad, CA, USA) and antibiotics and antimycotic (Lonza, Basel, Switzerland). Rhesus rotavirus (RRV) strain was kindly provided by Harry Greenberg (Stanford, CA, USA), and human rotavirus (HRV) strain ST-3 genotype G4P [6] was kindly provided by David Allen (Public Health England, UK). Working stocks were obtained by infecting MA104 cells at a multiplicity of infection (MOI) of 0.1 in MEM supplemented with 0.5 μg/mL trypsin IX (Sigma), and harvesting at 100% cytopathic effect.

### 2.2. Sequence-Independent Cloning of RRV Segments

DLPs were purified from RV-infected cells [24], and complementary DNA (cDNA) copies of 11 genomic segments were amplified from viral dsRNA, using a sequence-independent method [25]. Each segment was cloned, fully sequenced and engineered with a T7 promoter inserted preceding the first nucleotide of each segment. Restriction enzymes BbsI (S2, S8 and S10) and BsmBI (S1, S3, S4, S5, S6, S7, S9, S11) were inserted at the 3′ ends to generate exact ends. For the RNA–RNA interaction assay, linearized T7 plasmids were used to synthesize S8 (1059 nts), S9 (1062 nts), S10 (751 nts) and S11 (667 nts) RNA segments.

### 2.3. Electrophoretic Mobility Shift Assay (EMSA) for RNA–RNA Interaction

RNA segments were co-transcribed using 150 ng of linearized plasmid (S8–S11) in a buffer containing 40 mM Tris-HCl (pH 7.5), 10 mM MgCl_2_, 20 mM NaCl_2_, 3 mM spermidine, 50 mM dithiothreitol (DTT), 5 mM each of rATP, rUTP, rCTP, and rGTP, 10 U of RNase inhibitor and 40 U of T7 RNA polymerase (Thermo Scientific, Waltham, MA, USA). The mixture was incubated for 3 h at 37 °C, followed by RNase-free DNase One treatment. The reaction was immediately analyzed by electrophoresis in a 1% agarose gel supplemented with 0.1 mM MgCl_2_ in a TBM buffer (45 mM Tris-HCl (pH 8.3), 43 mM boric acid, and 0.1 mM MgCl_2_) at 160 V and 4 °C for 2.5 h, followed by staining with 0.01% (*w*/*v*) ethidium bromide. The integrity of the transcribed RNA was checked by denaturing gel electrophoresis on a 1% agarose gel with formaldehyde (10 mL of 10× MOPS running buffer) and 18 mL of 37% formaldehyde (12 M) on a pH 7.0 1× MOPS running buffer (0.4 M MOPS, 1 M sodium acetate, and 0.01 M ethylenediaminetetraacetic acid (EDTA)). For quantitative reverse transcription (qRT)-PCR analysis of heterodimeric RNAs, the shifted bands were extracted from excised gel using the NucleoSpin Gel and PCR Clean-up kit (Macherey-Nagel, Düren, Germany) following the manufacturer’s procedure.

The RNA–ORN hybridization assay was carried out by the 3′ end labeling of 10 pmol of S10 and S11 ORNs (10.2, 10.3 and 11.2 ORNs) including scrambled (Scr) with 10 μCi [^32^P]pCp (Perkin Elmer, Waltham, MA, USA) with T4 RNA ligase (Thermo Scientific, Waltham, MA, USA) in a T4 RNA ligase buffer, and incubating at 4 °C overnight. Unincorporated ^32^P was removed by exclusion chromatography (Illustra Microspin G-25 column, GE Healthcare, Chicago, IL, USA). Unlabeled S10 and S11 RNAs were denatured at 80 °C for 1 min, immediately chilled, and then mixed with a folding buffer (50 mM sodium cacodylate, pH 7.5; 100 mM KCl; and 10 mM MgCl_2_). Hybridization was performed with 1 pmol of pre-folded S10 RNA annealed with ^32^P-labelled ORNs (1, 2 or 4 pmol of ORNs) in the folding buffer in a 10 µL final volume [26]. The complex was allowed to form for 30 min at 30 °C, followed by electrophoresis in 4% native acrylamide gel at 150 V and 4 °C for 50 min in TBM buffer, dried and exposed by autoradiography.

### 2.4. Design of Antisense Oligoribonucleotides Based on Predicted ssRNA Structures

A series of eight antisense ORNs were designed to hybridize to the AUG initiating codon, stop codon and the 3′-UTRs of segments S1, S10 and S11. ORNs were modified at the 2′-OH ribose with a 2′-O-methyl group (Integrated DNA Technologies, Coralville, IO, USA) to enable nuclease resistance and to avoid potential interferon responses [27,28,29]. The Scr sequence was included as a specificity control, and was verified by NCBI-BLAST software [30] to prevent any match in the RV or HRV genomes or the host cellular RNAs. For the design of the ORN target sites, the software Mfold [31] and RNAfold [32] were used to predict the secondary structure and folding pattern of each RNA segment. To avoid structures that could prevent base pairing to target RNA, OligoAnalyzer [33] was used [27,29,34].

### 2.5. Oligoribonucleotide Interference Assay

For the ORN interference assay, the simultaneous transcription of two or more RNA segments (S8–S11) was performed in the presence of 20 pmol of S10 ORNs (S10.2 and S10.3) or S11 ORNs (S11.1 and S11.2), after determining the optimum inhibitory concentration of 20 pmol. The reactions were analyzed in 1% native gel electrophoresis, as described above, and the integrity of transcribed RNAs was verified by denaturing gel electrophoresis. For the RNA–RNA interaction specificity assay, co-transcription of S9–S11 was carried out in the presence of a 20-fold molar excess of transfer RNA (tRNA).

### 2.6. Virus Replication Inhibition Assay

For the inhibition of RV replication in the presence of ORNs, MA104 cells in 12-well plates (~5 × 10^5^ cells) were transfected with 0.5, 1, 1.5 or 3 µM ORNs for 3 h prior to infection with RV (MOI: 0.1) for 1 h. After 16–18 h, cells were harvested and the virus titer was analyzed by a plaque assay. For the time course assay, the experiment was repeated using 1.5 µM ORNs, and transfected-infected cells were harvested at different time points. RV genome copy numbers were determined by qRT-PCR, detecting the negative-strand RNA as described below.

### 2.7. Mutagenesis of RV Segment S10

Two S10 RNA mutations were generated, targeting the position of the S10.2 (27 nts) ORN by site-directed mutagenesis [35]. For the deletion of the mutant 10.2del (nt 570–596), S10 specific primers (forward 5′-GACTGCATCATTGTGAGGAAGCGGCGGAGTTCTTAAC-3′ and reverse 5′-GTTAAGAACTCCGCCGCTTCCTCACAATGATGCAGTC-3′) were used, and for the substitution, mutant 10.2mut (forward 5′-GCATCATTGTGATCGTGCAATACTAGGATCACTATTACAGGAAGCGGCGGAG-3′ and reverse 5′-CTCCGCCGCTTCCTGTAATAGTGATCCTAGTATTGCACGATCAAATGATGC-3′) was used.

### 2.8. Agarose Beads Pull-Down RNA–RNA Interaction Assay

The Agarose beads-based RNA–RNA interaction assay was described previously [19]. The biotin-labelled primer used for S11 coating was Rota S11R btn: 5′-[btn]AAAAAAAAAGAAATCCACTTGATCGCATCCAACGTTACTTG-3′. The coated beads were incubated with different sets of RNA in an RNA interaction buffer (50 mM sodium cacodylate, pH 7.5; 300 mM KCl; and 10 mM MgCl_2_) for 30 min at 30 °C, followed by three washes. The pulled-down RV S1 or S5 were quantified by qRT-PCR.

### 2.9. qRT-PCR

For the detection of ssRNA in RNA–RNA interactions and ORNs inhibition assays, ssRNAs were reverse-transcribed into cDNA using RevertAid Reverse Transcriptase (Thermo Scientific, Waltham, MA, USA) with specific reverse primers, and quantified with qPCR using the 7500 Fast Real-Time PCR system and SYBR select Master Mix (Applied Biosystems, Foster City, CA, USA). The primers were: for RRV S1: 2356F: 5′-AGAGTATGGGACAACTGACG-3′; RRV S1: 2570R: 5′-CACTTGAGAAAAGACGT GCGCAAATATCAGC-3′; RRV S5: q-rota S5F: 5′-CTGATCTCGGACTCTG-3′ and rota S5R: 5′-GGTCACAGTTTTTG-3′; RRV S8: q-rota S8F: 5′-GGAAAGTAATCCG-3′ and rota S8R: 5′-GGTCACATAAGCGC-3′; RRV S9: q-rota S9F: 5′-GTAGTCGACTATGTG-3′ and rota S9R: 5′-GGTCACATCATAC-3′; RRV S10: q-rota S10F: 5′-CATCGGACCTGATG-3′ and rota S10R: 5′-GGTCACAGGAAGAC-3′; and RRV S11: q-rota S11F: 5′-CTGTAAATATAAG-3′ and rota S11R: 5′-GGTCACAAAACGGAAGTGG-3′. The PCR conditions were 95 °C for 15 s and 55 °C for 30 s, for 40 cycles. The qPCR amplification was considered valid only when the standard curves had a correlation coefficient greater than 0.96 and a PCR efficiency within the range of 85–115%.

Since the de novo positive-strand RNA segments must have been associated with the newly synthesised viral capsid, prior to synthesis of negative-strand RNA of genomic dsRNA segments, viral dsRNA quantification was performed by using primers specifically targeting the negative strands in the RT reaction.

### 2.10. In Vitro Translation of RV Transcripts in the Presence of Oligoribonucleotides

For the in vitro RV translation assay, various concentration of ORNs RV S1 AUG (0.5, 1, 2 or 4 µM), S10.2, S11.2 (1 or 4 µM) or Scr (4 µM) were incubated with 300 ng of S1, S10 or S11 RV transcripts purified by phenol-chloroform-isoamyl alcohol, followed by exclusion chromatography (Illustra Microspin G-25 column, GE Healthcare, Chicago, IL, USA) to remove other impurities. The mixtures of RNA and ORN were incubated for 20 min at 37 °C, and added to a reaction mix containing 7.5 µL of nuclease-treated rabbit reticulocyte lysate (RRL, Promega, Madison, WI, USA), 1 mM amino acid mix minus methionine, and 6 µCi ^35^S-methionine. Translation reactions were incubated at 30 °C for 90 min and treated with 1 µg of RNase A for 10 min at room temperature. Labelled proteins were visualized using a PhosphorImager screen (GE Healthcare, Chicago, IL, USA).

## 3. Results

### 3.1. Determination of Intermolecular RNA–RNA Interactions between RRV Segments

**Figure 1:**
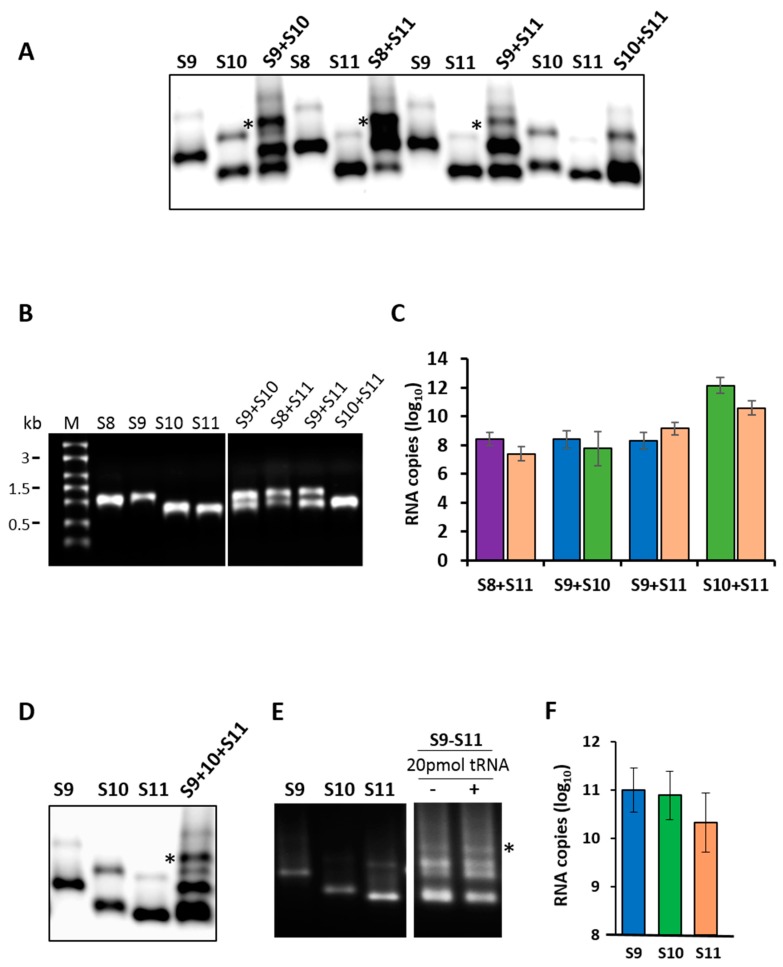
RNA–RNA interactions by the retardation assay. (**A**) Segments S8 to S11 were co-synthesized in combinations of two segments and analyzed by electrophoretic mobility shift assay (EMSA). Several retarded bands from the different RNA combinations are visible. Individually synthesized transcripts are shown as control. Retarded complex is marked with (*). (**B**) Co-transcribed or individual RNA transcription products of the same reaction used in the EMSA analysis were analyzed in a 1% denaturing agarose gel alongside the molecular size marker (M). (**C**) Detection of specific segments in retarded bands (*) shown in the EMSA analysis by quantitative reverse transcription (qRT)-PCR. The mean and standard deviations are shown (*n* = 3). (**D**) Segments S9–S11 were co-synthesized and the resulting RNA complexes were visualized by EMSA analysis. (**E**) Specificity of the interaction was tested in the presence (+) or absence (−) of tRNA. (**F**) The presence of specific segments in the retarded band (*) in (**D**) was verified by qRT-PCR. The mean and standard deviations are shown (*n* = 3).

To investigate if specific RNA–RNA interactions occurred among RV RNA segments, first, we synthesized exact copies of the small RNA segments (S8–S11) from the RV strain RRV by generating cDNA copies of genomic dsRNA segments from purified RV. Each cDNA copy was flanked by a T7 promoter at the 5′ terminus and a specific restriction enzyme site at the 3′ terminus, as described previously [36]. For RNA–RNA interactions, the synthesis of two or more small segments in different combinations was performed by co-transcription of cDNAs, simultaneously driven by the 5′ T7 promoter, as described previously [18,23]. The products of each reaction were analyzed on a 1% agarose gel by EMSA, to visualize the RNA–RNA complexes (Figure 1A). In parallel, an aliquot of each reaction was analyzed by denaturing agarose gel electrophoresis to ensure that ssRNA transcripts were synthesized in the mixtures with correct sizes (Figure 1B). Distinct shifted RNA bands of a higher molecular weight were detected by EMSA in co-transcribed reactions of different segment combinations (Figure 1A). For pairs S8 + S11, S9 + S10 and S9 + S11, we observed bands with higher molecular masses than those corresponding to the monomeric RNA or homodimer by EMSA, suggesting that specific RNA–RNA interactions occurred between the smaller RNAs to form RNA complexes with higher molecular masses. Since the homodimeric band of both segments S10 and S11 had a similar mobility to the heterodimers in the native gel, it was not possible to decipher the specific RNA–RNA interactions between these two segments by EMSA. Therefore, bands were excised and a segment-specific qRT-PCR analysis was performed, which demonstrated that both segments S10 and S11 were present in the excised band (Figure 1C). The integrities of the co-synthesized and individual RNA segments were also validated by denaturing agarose gel electrophoresis, showing the intact RNA segments.

To further investigate the interactions among all three segments (S9, S10 and S11), ssRNA of each segment was co-synthesized and analyzed by EMSA (Figure 1D). Whilst homodimers were observed, there was a shift towards the formation of heterodimeric RNA complexes. This suggested the requirement for another segment in order to form a more stable complex. Subsequently, we confirmed and quantitated the presence of each segment in the heterodimeric complexes by segment-specific qRT-PCR assays (Figure 1F). The specificity of the observed interactions was investigated in parallel, using an RNA–RNA interaction assay, which was performed in the presence of 20-fold molar excess of non-specific competitor yeast tRNA. No disruption of the RV RNA complex formation was observed, indicating that RNA–RNA interactions between the RV segments were specific (Figure 1E).

### 3.2. Pull-Down Assay Suggests Networking between RNA Segments of Different Sizes

The mechanism by which viruses with multi-segmented dsRNA genomes correctly package the complete set of genomic RNA segments is not fully understood. Our previous study on BTV demonstrated that the genomic RNA segments interact with each other in a coordinated manner that is related to the segment size and is initiated by the small segments, which leads to a sequential packaging mechanism [19]. We predicted that RV adopted a similar mechanism for genome assembly. Our EMSA data (above) indicated that, similarly to BTV, the smaller segments of RV interact with each other and form complexes, and it is predicted that the 3′-UTRs of RVs are similar to another member of the *Reoviridae* family—the orbiviruses—where the 3′-UTRs of the RNA segments play an important role in RNA complex formation [6,7,21,22,23,24,25]. However, due to the limitation of the EMSA method, it was not possible to visualize the complex forming by larger RNA segments in agarose gel. Therefore, it was necessary to establish a bead-based RNA–RNA interaction assay to detect the complex formation between smaller and larger RNA segments. We used a pull-down assay in which streptavidin beads were coated with S11, and different ssRNA segments were incubated with the coated beads, as described previously [19]. The RNA complexes captured by the beads were then detected by qRT-PCR using segment-specific primers. RNA pulled-down by non-coated beads served as the baseline for quantification of ssRNA pulled-down by S11.

**Figure 2:**
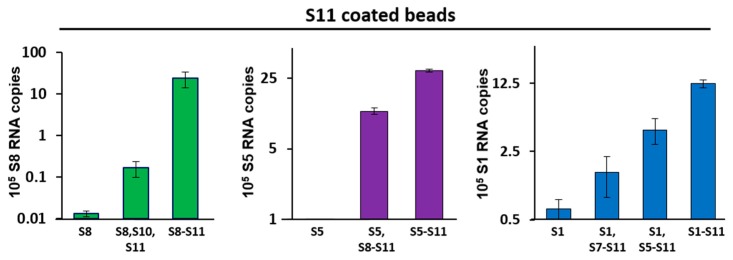
RNA–RNA interactions between smaller and larger segments by pull-down assays. Agarose beads coated with S11 were incubated with different sets of RNA segments that included small (**left** panel), medium (**centre** panel) and large (**right** panel) size segments. After extensive washing of the beads, RNA–RNA interactions were detected by qRT-PCR using primers specific for the interacting segment: S8 (left), S5 (centre) or S1 (right). RNA pulled-down by uncoated beads was set as the baseline. The mean and standard deviations are shown (*n* = 3).

Our data show that S11-coated beads captured S8 efficiently in the pull-down assay when S9 and S10 were present, but this was 100-fold less when only S10 was added in absence of S9 (Figure 2; left panel). Similarly, S11-coated beads pulled-down S5 efficiently, but only when segments S6–S10 were present; removing segments S6 and S7 from this set decreased the binding by 3-fold. S5 alone did not bind to S11-coated beads (Figure 2; middle panel). Furthermore, S1 was pulled-down efficiently when all other nine segments (S2–S10) were present (Figure 2; right panel). S1 had a lower level of interaction with segments S5–S11 in the absence of the other three large segments (S2–S4). S1 had a very low affinity with S11-coated beads in the absence of other segments or if only smaller segments were involved. These data suggested that, similar to orbiviruses, multi-segment RNA complex networks might play a role in the RV genome assembly.

### 3.3. ssRNA Segment Interactions and Their Interruptions by Specific Oligoribonucleotides

**Figure 3:**
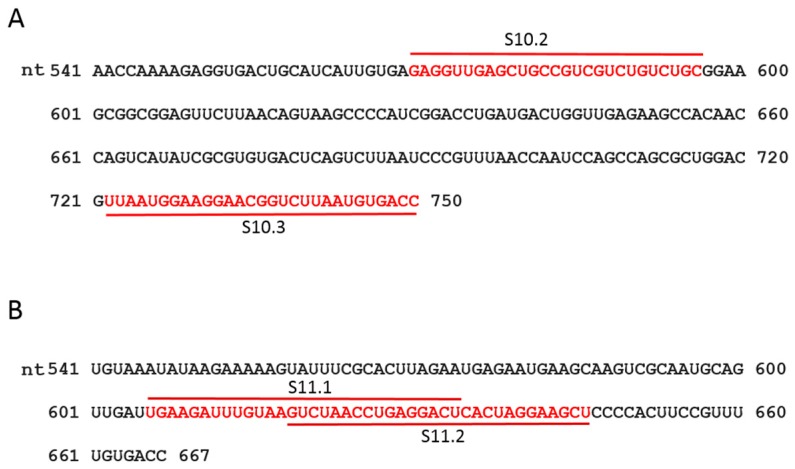
Sequence and position of designed ORNs. The sequence of 3′-UTR of segments S10 (**A**) and S11 (**B**) are shown. The target positions of the designed ORNs are indicated in red.

**Figure 4:**
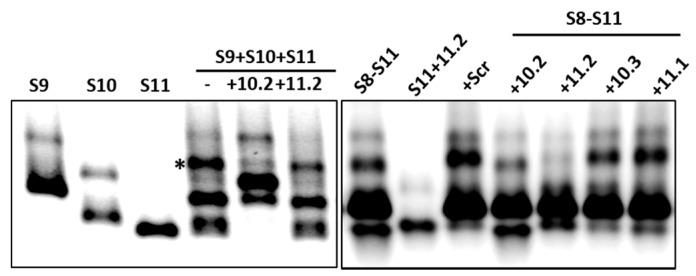
Effects of ORNs on RNA–RNA interactions. Combinations of three (S9–S11) or four (S8–S11) RNA segments were co-transcribed in the presence of S10 or S11 ORNs as indicated and analyzed by EMSA. Individually transcribed segments are shown as control. Retarded complex is marked with (*).

It has previously been demonstrated that short ORNs targeting the 3′-UTR of the smallest RNA segment of BTV can perturb RNA–RNA interactions and the RNA complex formation [18]. We investigated whether short, specific antisense ORNs targeting the 3′ terminal sequences of the smaller segments of RRV would interfere with RNA–RNA interactions and disrupt the formation of RNA network complexes. To this end, a series of short (27–29 nts) nuclease-resistant ORNs were designed (Table 1 and Figure 3A,B) to base pair with the 3′-UTR of segments S10 or S11. In particular, these were designed to target the single-stranded region, bulges, and hairpin loops based on the RNAfold secondary structure prediction of the S10 and S11 RNA, as no structural data was currently available for RV RNA segments. These regions have also been reported to be important for dsRNA virus RNA–RNA interactions and genome packaging [10,14,18,19,37]. In the presence of target ORNs, or a scrambled control derivative (Scr ORN), co-transcription reactions of the three and four segment combinations (S9–S11 and S8–S11) were performed, and the products were analyzed by EMSA. The effects of ORNs on RNA–RNA interactions and the complex formation were clearly visible, particularly on the three smallest RRV segments (S9 + S10 + S11), showing a reduction of higher molecular mass bands representing heterodimer/RNA complexes in the presence of ORN 10.2 (Figure 4). However, less inhibition in RNA–RNA interactions was observed in the presence of ORN 11.2, which suggested that each ORN affected their target RNAs at different levels. The appearance of an additional band, which corresponded to the size of the S10 dimer, may have suggested a conformational change in the S10 RNA by forming a hybrid with ORN 10.2 and blocking its interactions with other segments.

To investigate if the same ORNs could inhibit the RNA complex formation in the presence of additional segments, S8 was added to the S9–S11 combinations and co-synthesized in the presence of ORNs 10.2 or 11.2, and also with two additional ORN-targeting 3′-UTRs (S11.1 and S10.3). The results obtained indicated that S11.2 inhibited the RNA complex formation or RNA–RNA interactions more efficiently than the S10.2 ORN in S8–S11 RNA combinations (Figure 4), suggesting a differential effect of the ORNs in blocking the interacting sites to make RNA–RNA contacts. The ORNs 10.3 and 11.1 had modest or no effects on the RNA complex disruption (Figure 4), suggesting these regions of the 3′-UTRs were either not involved or were less involved in RNA–RNA interactions. No significant reduction of the complex formation was observed in the reaction with the non-specific Scr control ORN (Figure 4).

**Figure 5:**
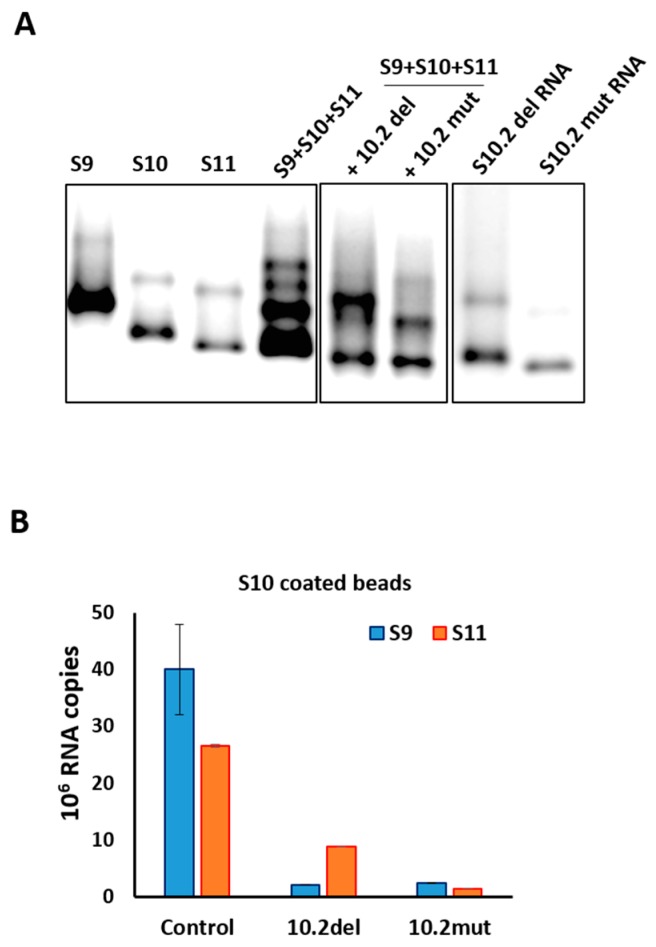
RNA–RNA interaction with S10 mutants by the retardation assay. (**A**) Segments S9–S11 were co-synthesized in the presence of S10 RNAs with deletion (10.2del) or substitution (10.2mut) mutations. Wild-type S10 and individually transcribed segments are shown as control. Retarded bands are visible on co-transcribed lanes. (**B**) Pull-down assay with agarose beads coated with segments 10.2del, 10.2mut or wild type (control). S9 and S11 were detected in each experiment by qRT-PCR using specific primers. The mean and standard deviations are shown (*n* = 3).

To further confirm and substantiate the observed disruption on the RNA complex formation using S10.2 ORN, two mutations were designed on S10 RNA, one with a deletion (10.2del) and one with substitution mutation (10.2mut) spanning the same targeted area. The region of deletion mutation was the 27 nts (nt 570–596) after the NSP4 stop codon (5′-GAGGUUGAGCUGCCGUCGUCUGUCUGC-3′), complementary to S10.2 as shown in Table 1. For the substitution mutation, the same sequence was substituted to 5′-UCGUGCAAUACUAGGAUCACUAUUACA-3′ to disrupt the predicted secondary structure. When the S10 deletion or substitution mutants were used in the RNA–RNA interaction of S9 + S10 + S11, there was significant disruption in the RNA complex formation analyzed by EMSA, suggesting that this region was likely to be the binding site of S10 with other segments (Figure 5A). The EMSA data were further confirmed by a pull-down assay, where wild-type and mutant S10-coated beads were used to pull-down S9 and S11. In the presence of either ORN S10.2 or S11.2, both S9 and S11 were captured by pull-down significantly less efficiently, especially S9. Similarly, when mutant S10-coated beads were used, the recovery of segments S9 and S11 by pull-down were reduced (Figure 5B).

### 3.4. Inhibition of Virus Replication with Oligoribonucleotides 3′-UTRs

After demonstrating the capacity of the ORNs to inhibit the RNA complex formation in vitro, we investigated whether the inhibition of RNA–RNA interactions mediated by ORNs affected RV replication in cell culture. Since the ORNs were modified at the 2′-ribose to substitute the 2′-OH to 2′-O-methyl, these small ORNs should not have had any adverse cellular interferon effects during the virus replication [27,28,29]. The optimization of ORN concentrations for cell toxicity was first determined, and subsequent virus replication assays were performed in the presence of 1.5 µM ORN, which was determined to be the optimum concentration.

**Figure 6:**
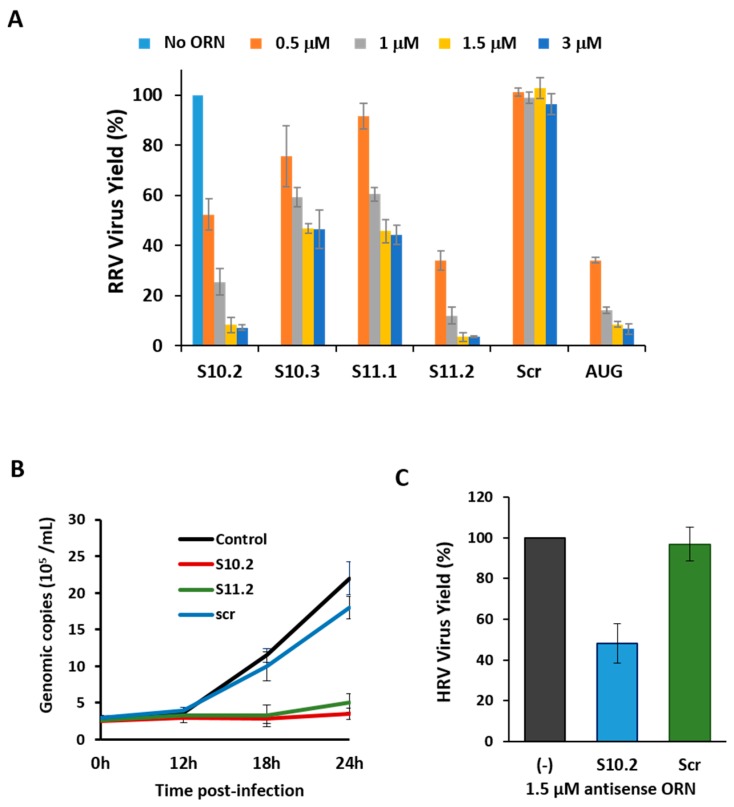
Effect of ORNs on virus replication. (**A**) Quantification of plaques (in percentage) in the presence of increasing amounts (500 nM to 3 µM) of different ORNs, relative to the control (no ORN). The mean and standard deviations are shown (*n* = 4–6). (**B**) Genome copy number determined by qRT-PCR in the presence (S10.2, S11.2 or Scr) or absence (control) of ORNs at indicated times post-infection. (**C**) Quantification of plaques (in percentage) of human rotavirus (HRV) in the presence or absence (−) of rhesus rotavirus (RRV) ORN (S10.2 or Scr), relative to the control. The mean and standard deviations are shown (*n* = 3).

Scr ORN and an S1 ORN (Table 1) complementary to AUG-initiating codon sequences of RV VP1 polymerase protein [1,2] were used as controls. All ORNs targeting segments S10 and S11 had some inhibitory effects on the virus growth, to varying degrees, with ORNs S10.2 and S11.2 exhibiting potent inhibitory effects (Figure 6A). The ORN S10.2 targeted the 27 nts sequence after the stop codon of the NSP4 gene, and ORN S10.3 complemented the extreme 29 nts on the 3′-UTR (181 nts), including the conserved terminal sequence (see Figure 3A). The ORN S11.2, which had the most inhibitory effect on the virus replication, was complementary to the 29 nts downstream of the NSP5 gene stop codon, up to the 3′-UTR terminus. The ORN S11.1, which had a reduced effect on the inhibition of RV replication, encompassed 29 nts upstream from the stop codon. The inhibitory effects of ORNs on the viral replication suggested that 3′-UTR sequences of these two smallest segments were crucial for the virus replication.

To further substantiate the inhibitory effects of the S10.2 and S11.2 ORNs on the virus growth, we measured the dsRNA synthesis in the presence or absence of these two ORNs at different time points of the viral replication. Since RV only produces dsRNA as genomic RNA segments, and each viral particle contains precisely one copy of each of the 11 genomic dsRNA segments, the copies of dsRNA segments represented the quantity of the viral genome present in the infected cells. Quantitative analysis of viral dsRNA using qRT-PCR confirmed that S10.2 and S11.2 significantly inhibited the virus replication (Figure 6B).

The nucleotide sequences of the RRV 3′-UTR shared some similarity with those of human RV (HRV), and since the 3′-UTR of segment S10 was the longest among all segments, we assessed the effect of the S10.2 ORN (nt 570–596) on the replication of an HRV strain in MA104 cells. The S10.2 ORN corresponding region of the HRV S10 segment had only two mismatches in comparison to that of RRV. The inhibition of the virus growth was at least ~50% in the presence of S10.2 (nt 570–596; RRV/HRV; Figure 6C). These common inhibitory effects of the ORN indicated that the critical region for HRV replication must have been targeted by the ORN, despite the differences in the sequence.

**Figure 7:**
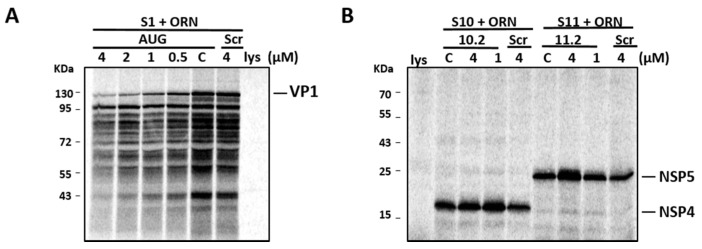
RRV mRNA translation efficiency in vitro in the presence of ORNs. In vitro synthesized VP1 (**A**) in the absence (c) or presence of S1 AUG or Scr ORNs, and NSP4 and NSP5 (**B**) in the absence (c) or presence of S10.2 or S11.2 ORNs, in varying concentrations (μM). As the negative control, rabbit reticulocyte lysate (lys) is shown. Positions of molecular mass standards are indicated in kDa.

To ensure that the reduced virus replication was not due to the inhibition of viral protein synthesis, we performed an in vitro translation of viral proteins in the presence and absence of S10.2 and S11.2 ORNs. In parallel, the S1 AUG ORN (as an inhibitory control) and Scr ORN (as a non-inhibitory control) were included. Figure 7A shows the dose-dependent inhibitory effects of the S1 AUG ORN control on in vitro VP1 synthesis, where inhibition was observed from 0.5 µM up to 4 µM, while no inhibition of the VP1 synthesis could be detected in the presence of Scr ORN. In contrast, even at the highest concentration of ORN (4 µM), the ORNs targeting S10 and S11 RNA did not inhibit the synthesis of NSP4 and NSP5 proteins, respectively. As expected, there was no detectable inhibition of these proteins in the presence of Scr ORN (Figure 7B). These results suggested that the 3′-UTR-targeting ORNs, although inhibitory in the viral replication assay, had no effect on the viral protein synthesis.

**Figure 8:**
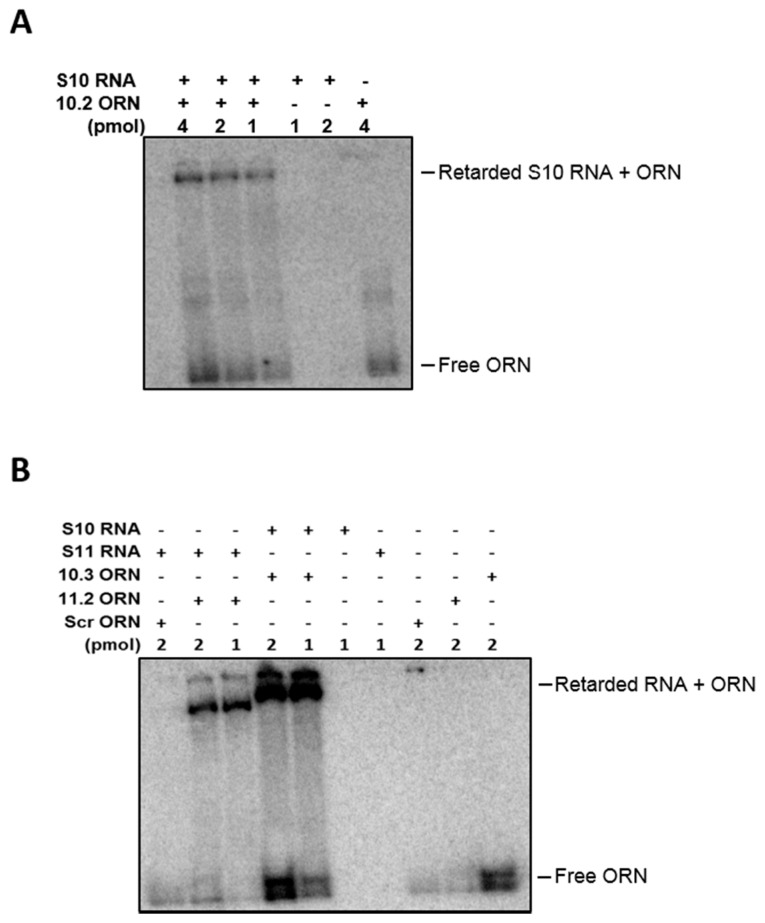
Hybridization assay between ORNs and target RNAs. (**A**) ^32^P-labelled S10.2 (1, 2 and 4 pmol) ORNs were hybridized to 1 pmol of S10 in a folding buffer and incubated for 30 min at 30 °C. The hybrids were analyzed on 4% native acrylamide gel, followed by autoradiography. (**B**) ^32^P-labelled S10.3, 11.2 and Scr (1 and 2 pmol) ORNs were hybridized to 1 pmol of S10 or S11 in a folding buffer and incubated for 30 min at 30 °C. The hybrids were analyzed on 4% native acrylamide gel, followed by autoradiography.

To further investigate the specificity of the ORNs to the target regions, hybridization assays of RNAs and ORNs were undertaken. The gel analysis showed that ORNs S10.2, S10.3 hybridized with S10 and S11.2 hybridized with S11 mRNA. No hybridization with the Scr control was detected when incubated with S11 RNA, suggesting specific annealing of the ORNs to their target sequences (Figure 8).

## 4. Discussion

The precise and specific mechanism of genome segment assembly during RV replication has yet to be elucidated. Previously, our laboratory has reported that BTV hierarchical intermolecular interactions between RNA segments are required for the efficient packaging of the viral genome [13,18,19,23]. It was hypothesized that members of the same family, such as RV, may employ the same mechanism of genome assembly. We therefore established a number of in vitro assay systems to investigate the formation and possible disruption of RNA–RNA interactions. Using the EMSA method, we demonstrated that the small RV RNA segments make specific interactions. Although the quantities of cDNA of each segment used in all experiments were equal, there were small variations in the RNA synthesis in some reactions. However, RNA complexes were still easily detectable between two different segments in each combination tested, indicating that interactions between different transcripts occurred during transcription. The RNA complexes were also visible when additional segments were added to the combinations (3–4 segments in a single transcription reaction). This indicated that stronger interactions between different RV genome segments likely required an additional segment to form stable RNA complexes, as previously proposed for BTV [14,18,19].

Since EMSA is not appropriate for the detection of very large complexes, an alternative pull-down assay using the immobilized RV segment S11 was established to evaluate the interactions between the larger and smaller genome segments. The smaller genome segments appeared only to interact with the larger genome segments (such as S1) via the medium-sized RNA segments. The medium-sized RNA segment S5 also required the presence of other intermediate-sized segments (S6–S10) in order to interact with S11. These data suggest that all 11 RV genome segments may form a networking RNA complex. We predict that the formation of an RNA complex comprised of the small RNA segments serves as the leading base for recruiting the remaining RNA segments by sequential interactions, and ultimately generating a complex composed of the complete set of 11 RV RNA segments, similarly to for BTV [18,19].

Based on previous studies of BTV, we proposed that the 3′-UTR of smaller segments might have also played an important role in RV genome assembly. Our data showed that intersegment interactions between the smaller segments could be disrupted in the presence of ORNs specifically targeting the 3′-UTR of segments S10 and S11, as demonstrated by altered RNA migration profiles on native agarose gels. The different effects of the S10- and S11-targeting ORNs on the complex formation were most likely due to the different secondary structures and contact points disrupted by each specific ORN. Deletion and substitution mutation in the S10 3′-UTR region targeted by the ORNs significantly disrupted the RNA complex formation, also suggesting that this region was likely to be the putative binding sites of S10 with other genome segments. In summary, our data suggest that both ORNs and specific mutations in the 3′-UTR of genome segments, are able to disrupt the specific RNA structures in the 3′-UTR, resulting in conformational changes that affect the correct sorting and/or assembly of RV genome RNA segments.

This hypothesis was substantiated by demonstrating that the ORNs, which caused disruption of RNA–RNA interactions in a RRV strain in vitro, also had strong inhibitory effects on RV replication in cell culture, using a HRV strain. This suggests that ORNs targeting the 3′-UTR of RV RNA genome segments might cause the same inhibitory effect on RV replication of the two related RV species. The observed inhibition of RV replication was likely to be due to disruption in the genome sorting and/or packaging process, as the presence of these ORNs did not inhibit the virus protein expression. Some ORNs significantly inhibited the virus growth, but had mild effects on the RNA interaction. As the interactions may depend on multipoint contacts between RNA segments, blocking one region of an RNA segment will not necessarily fully diminish the complex formation in vitro, however, it is sufficient to prohibit the assembly of all RNA segments and consequently abolish the virus replication in vivo. Further RNA structural analysis is needed to confirm our hypothesis.

Most current antiviral agents target specific viral enzymes, such as polymerases and proteases, and these often become ineffective after long-term use, as the target viruses develop a resistance to the drug. To minimize the possibility of antiviral drug resistance, it might be a useful strategy to target other stages in the virus replication process, such as viral genome packaging mechanisms possibly targeting specific sequences of the conserved 3′-UTRs [11,38,39]. Our demonstration here of both the intermolecular RNA–RNA interactions and the disruption of these interactions using ORNs, both in vitro and in virus replication, illustrates a possible target for interfering with virus assembly. As genome assembly is a universal property of all RV strains, this mechanism may present an Achilles’ heel for RV, towards a better understanding of genome sorting and packaging amongst RV species and other members of the *Reoviridae* family.

## Figures and Tables

**Table 1:** Sequence and position of designed oligoribonucleotides (ORNs). The length of the ORN is shown in brackets and the target position in each segment is indicated in the right column.

ORN	Sequence (2′-*O*-Methyl Modified)	Position
S1 AUG	5′-GAUUAGAUUAUACUUCCCCAUUGUAUAGC-3′ (29 nts)	28–56
S10.2	5′-GCAGACAGACGACGGCAGCUCAACCUC-3′ (27 nts)	570–596
S10.3	5′-GGUCACAUUAAGACCGUUCCUUCCAUUAA-3′ (29 nts)	722–750
S11.1	5′-AGUCCUCAGGUUAGACUUACAAAUCUUCA-3′ (29 nts)	606–625
S11.2	5′-AGCUUCCUAGUGAGUCCUCAGGUUAGAC-3′ (28 nts)	619–646
Scr	5′-UCGUGCAAUACUAGGAUCACUAUUACAU-3′ (28 nts)	-
